# Methyl 3-[(*E*)-1-(4-amino­phen­yl)ethyl­idene]dithio­carbazate

**DOI:** 10.1107/S160053680801283X

**Published:** 2008-05-07

**Authors:** Shang Shan, Shan-Heng Wang, Yu-Liang Tian, Wen-Long Wang, Ying-Li Xu

**Affiliations:** a College of Chemical Engineering and Materials Science, Zhejiang University of Technology, People’s Republic of China

## Abstract

The title compound, C_10_H_13_N_3_S_2_, was obtained from a condensation reaction of methyl dithio­carbazate and 4-amino­acetophenone. In the crystal structure, the nearly planar mol­ecule assumes an *E* configuration, the benzene ring and dithio­carbazate group being located on opposite sides of the N=C bond. C—H⋯π inter­actions and N—H⋯S hydrogen bonding are present in the crystal structure.

## Related literature

For general background, see: Okabe *et al.* (1993 ▶); Shan *et al.* (2003 ▶); Jiang (2007 ▶). For related structures, see: Shan *et al.* (2006 ▶); Zhang *et al.* (2005 ▶). For synthesis, see: Hu *et al.* (2001 ▶).
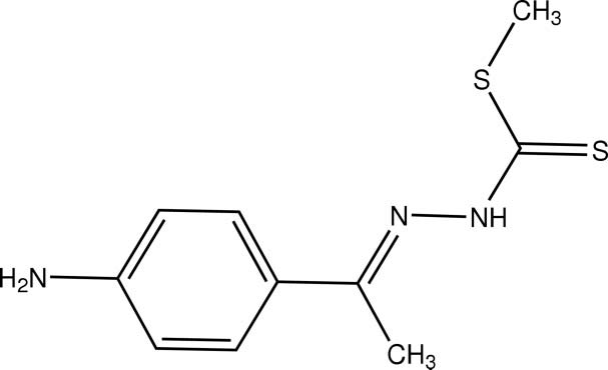


## Experimental

### 

#### Crystal data

C_10_H_13_N_3_S_2_
*M* *_r_* = 239.35
Monoclinic, 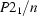

*a* = 10.8247 (12) Å
*b* = 5.3673 (8) Å
*c* = 20.4549 (14) Å
β = 94.756 (12)°
*V* = 1184.3 (2) Å^3^
*Z* = 4
Mo *K*α radiation
μ = 0.42 mm^−1^
*T* = 295 (2) K
0.32 × 0.22 × 0.20 mm

#### Data collection

Rigaku R-AXIS RAPID IP diffractometer
Absorption correction: multi-scan (*ABSCOR*; Higashi, 1995 ▶) *T*_min_ = 0.870, *T*_max_ = 0.926
10489 measured reflections
2682 independent reflections
1867 reflections with *I* > 2σ(*I*)
*R*_int_ = 0.030

#### Refinement

*R*[*F* ^2^ > 2σ(*F* ^2^)] = 0.043
*wR*(*F* ^2^) = 0.124
*S* = 1.07
2682 reflections
138 parameters
H-atom parameters constrained
Δρ_max_ = 0.32 e Å^−3^
Δρ_min_ = −0.35 e Å^−3^

### 

Data collection: *PROCESS-AUTO* (Rigaku, 1998 ▶); cell refinement: *PROCESS-AUTO*; data reduction: *CrystalStructure* (Rigaku/MSC, 2002 ▶); program(s) used to solve structure: *SIR92* (Altomare *et al.*, 1993 ▶); program(s) used to refine structure: *SHELXL97* (Sheldrick, 2008 ▶); molecular graphics: *ORTEP-3 for Windows* (Farrugia, 1997 ▶); software used to prepare material for publication: *WinGX* (Farrugia, 1999 ▶).

## Supplementary Material

Crystal structure: contains datablocks I, global. DOI: 10.1107/S160053680801283X/om2229sup1.cif

Structure factors: contains datablocks I. DOI: 10.1107/S160053680801283X/om2229Isup2.hkl

Additional supplementary materials:  crystallographic information; 3D view; checkCIF report

## Figures and Tables

**Table 1:** Hydrogen-bond geometry (Å, °)

*D*—H⋯*A*	*D*—H	H⋯*A*	*D*⋯*A*	*D*—H⋯*A*
N1—H1*A*⋯S2^i^	0.90	2.83	3.722 (3)	170
N3—H3*N*⋯S2^ii^	0.94	2.59	3.483 (2)	159
C10—H10*A*⋯*Cg*^iii^	0.96	2.80	3.538 (3)	134

Symmetry codes: (i) 
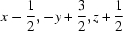
; (ii) 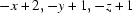; (iii) 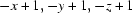. *Cg* is the centroid of the benzene ring.

**Fig. 1.**
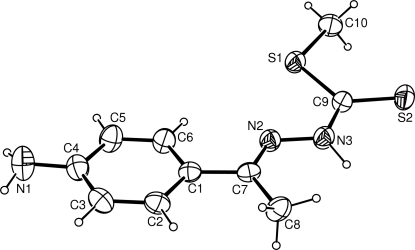
The molecular structure of the title compound with 30% probability displacement ellipsoids (arbitrary spheres for H atoms).

**Fig. 2.**
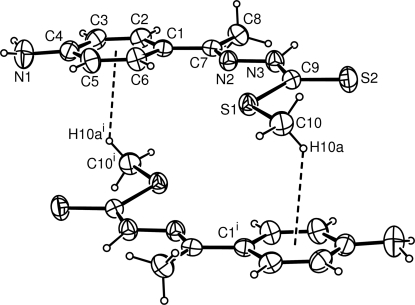
A diagram showing C—H···π interaction by dashed lines [symmetry code: (i) = 1 - x,1 - y,1 - z].

## supplementary crystallographic information

### Comment

As some phenylhydrazone derivatives have been shown to be potential DNA-damaging or mutagenic agents (Okabe *et al.*, 1993), a series of new phenylhydrazone derivatives has been synthesized in our laboratory in past years, and several crystal structures of phenylhydrazone compounds have been determined by X-ray diffraction in order to research their structure-bioactivity relationship (Shan *et al.*, 2003). Recent investigation discovered that sulfur-containing hydrazone compounds are benefit to promote the bioactivities of hydrazone (Jiang, 2007). As part of our ongoing investigation on hydrazone compounds, the title compound with dithiocarbazate component has recently been prepared and its crystal structure is reported here.

The structure of the title compound is shown in Fig. 1. The N2—C7 bond distance of 1.281 (3) Å indicates a typical C=N double bond. The molecule adopts an E configuration about the C=N double bond. The molecule has a nearly planar structure. The C8 atom is well co-planar with the benzene ring with a small atomic deviation of 0.028 (4) Å from the phenylmethylene mean plane. The dithiocarbazate moiety is slightly twisted to the phenylmethylene plane with a dihedral angle of 13.4 (1)°. The shorter N3—C9 bond distance of 1.345 (3) Å implies the N3 atom involved in the electron delocalization in the dithiocarbazate moiety.

It is notable that the N3—C9—S1 bond angle of 113.26 (15)° is much smaller than 120° expected for a *sp*^2^ hybrid C atom and also much smaller than the corresponding N3—C9—S2 bond angle of 121.66 (16)°, which is similar to that found in related structures reported previously (Shan *et al.*, 2006; Zhang *et al.*, 2005).

Intermolecular C—H···π interaction is observed between C10-methyl group and the benzene ring of the adjacent molecule (Fig. 2), C10—H10*a*—*Cg*^i^ angle being 134° and C10···*Cg*^i^ and H10*a*···*Cg*^i^ separations being 3.538 (3) and 2.80 Å, respectively [where *Cg* is the centroid of the benzene ring and symmetry code (i) = 1 - *x*,1 - *y*,1 - *z*]. Molecules are also linked by intermolecular C—H···S hydrogen bonding (Table 1) to form the supra-molecular chain.

### Experimental

Methyl dithiocarbazate was synthesized in the manner reported previously (Hu *et al.*, 2001). Methyl dithiocarbazate (1.24 g, 10 mmol) and 4-aminoacetophenone (1.35 g, 10 mmol) were dissolved in ethanol (10 ml) and refluxed for 6 h. Yellow crystalline product appeared after cooling to room temperature. They were separated and washed with cold water three times. Single crystals of the title compound were obtained by recrystallization from a 2-propanol solution.

### Refinement

H atoms bonded to N atoms were located in a difference Fourier map and refined as riding in their as-found relative positions with *U*_iso_(H) = 1.2*U*_eq_(N). Methyl H atoms were placed in calculated positions with C—H = 0.96 Å and torsion angles were refined to fit the electron density, *U*_iso_(H) = 1.5*U*_eq_(C). Aromatic H atoms were placed in calculated positions with C—H = 0.93 Å and refined in riding mode with *U*_iso_(H) = 1.2*U*_eq_(C).

### Crystal data

**Table:**

C_10_H_13_N_3_S_2_	*F*_000_ = 504
*M**_r_* = 239.35	*D*_x_ = 1.342 Mg m^−^^3^
Monoclinic, *P*2_1_/*n*	Mo *K*α radiation λ = 0.71073 Å
Hall symbol: -P 2yn	Cell parameters from 4876 reflections
*a* = 10.8247 (12) Å	θ = 3.5–25.0º
*b* = 5.3673 (8) Å	µ = 0.42 mm^−^^1^
*c* = 20.4549 (14) Å	*T* = 295 (2) K
β = 94.756 (12)º	Prism, yellow
*V* = 1184.3 (2) Å^3^	0.32 × 0.22 × 0.20 mm
*Z* = 4	

### Data collection

**Table:**

Rigaku R-AXIS RAPID IP diffractometer	2682 independent reflections
Radiation source: fine-focus sealed tube	1867 reflections with *I* > 2σ(*I*)
Monochromator: graphite	*R*_int_ = 0.030
Detector resolution: 10.00 pixels mm^-1^	θ_max_ = 27.5º
*T* = 295(2) K	θ_min_ = 3.4º
ω scans	*h* = −14→14
Absorption correction: multi-scan(ABSCOR; Higashi, 1995)	*k* = −6→6
*T*_min_ = 0.870, *T*_max_ = 0.926	*l* = −26→26
10489 measured reflections	

### Refinement

**Table:**

Refinement on *F*^2^	Secondary atom site location: difference Fourier map
Least-squares matrix: full	Hydrogen site location: inferred from neighbouring sites
*R*[*F*^2^ > 2σ(*F*^2^)] = 0.043	H-atom parameters constrained
*wR*(*F*^2^) = 0.124	*w* = 1/[σ^2^(*F*_o_^2^) + (0.0597*P*)^2^ + 0.2088*P*] where *P* = (*F*_o_^2^ + 2*F*_c_^2^)/3
*S* = 1.07	(Δ/σ)_max_ = 0.001
2682 reflections	Δρ_max_ = 0.32 e Å^−^^3^
138 parameters	Δρ_min_ = −0.35 e Å^−^^3^
Primary atom site location: structure-invariant direct methods	Extinction correction: none

### Special details

**Table:**

Geometry. All e.s.d.'s (except the e.s.d. in the dihedral angle between two l.s. planes) are estimated using the full covariance matrix. The cell e.s.d.'s are taken into account individually in the estimation of e.s.d.'s in distances, angles and torsion angles; correlations between e.s.d.'s in cell parameters are only used when they are defined by crystal symmetry. An approximate (isotropic) treatment of cell e.s.d.'s is used for estimating e.s.d.'s involving l.s. planes.
Refinement. Refinement of *F*^2^ against ALL reflections. The weighted *R*-factor *wR* and goodness of fit *S* are based on *F*^2^, conventional *R*-factors *R* are based on *F*, with *F* set to zero for negative *F*^2^. The threshold expression of *F*^2^ > σ(*F*^2^) is used only for calculating *R*-factors(gt) *etc*. and is not relevant to the choice of reflections for refinement. *R*-factors based on *F*^2^ are statistically about twice as large as those based on *F*, and *R*- factors based on ALL data will be even larger.

### Fractional atomic coordinates and isotropic or equivalent isotropic displacement parameters (Å^2^)

**Table:**

	*x*	*y*	*z*	*U*_iso_*/*U*_eq_	
S1	0.64254 (5)	0.21562 (12)	0.48386 (3)	0.0615 (2)	
S2	0.88535 (6)	0.31459 (16)	0.42531 (3)	0.0800 (3)	
N1	0.3499 (3)	0.7090 (5)	0.79285 (13)	0.0946 (8)	
H1A	0.3635	0.8087	0.8281	0.114*	
H1B	0.3062	0.5773	0.8021	0.114*	
N2	0.72126 (16)	0.5566 (3)	0.57515 (8)	0.0535 (4)	
N3	0.80638 (17)	0.5371 (4)	0.52873 (9)	0.0581 (5)	
H3N	0.8845	0.6145	0.5342	0.070*	
C1	0.63507 (19)	0.7276 (4)	0.66547 (10)	0.0482 (5)	
C2	0.6266 (2)	0.9072 (4)	0.71383 (10)	0.0597 (6)	
H2	0.6850	1.0346	0.7178	0.072*	
C3	0.5336 (2)	0.9013 (5)	0.75625 (11)	0.0671 (6)	
H3	0.5298	1.0257	0.7876	0.081*	
C4	0.4458 (2)	0.7123 (5)	0.75263 (11)	0.0630 (6)	
C5	0.4553 (2)	0.5303 (5)	0.70511 (13)	0.0691 (6)	
H5	0.3980	0.4009	0.7017	0.083*	
C6	0.5470 (2)	0.5376 (4)	0.66329 (12)	0.0615 (6)	
H6	0.5509	0.4117	0.6323	0.074*	
C7	0.73087 (18)	0.7311 (4)	0.61800 (10)	0.0483 (5)	
C8	0.8278 (2)	0.9319 (4)	0.62278 (12)	0.0641 (6)	
H8A	0.8693	0.9357	0.5831	0.096*	
H8B	0.8870	0.8982	0.6593	0.096*	
H8C	0.7891	1.0901	0.6290	0.096*	
C9	0.78466 (19)	0.3662 (4)	0.48112 (10)	0.0539 (5)	
C10	0.6466 (2)	−0.0091 (5)	0.41878 (12)	0.0720 (7)	
H10A	0.6459	0.0760	0.3775	0.108*	
H10B	0.5753	−0.1157	0.4186	0.108*	
H10C	0.7206	−0.1074	0.4255	0.108*	

### Atomic displacement parameters (Å^2^)

**Table:**

	*U*^11^	*U*^22^	*U*^33^	*U*^12^	*U*^13^	*U*^23^
S1	0.0515 (3)	0.0747 (4)	0.0591 (3)	−0.0119 (3)	0.0090 (2)	−0.0129 (3)
S2	0.0592 (4)	0.1134 (6)	0.0703 (4)	−0.0144 (4)	0.0219 (3)	−0.0215 (4)
N1	0.0967 (18)	0.0956 (18)	0.0979 (17)	0.0018 (14)	0.0456 (14)	−0.0022 (15)
N2	0.0518 (10)	0.0545 (10)	0.0548 (9)	−0.0059 (8)	0.0083 (8)	−0.0056 (9)
N3	0.0511 (10)	0.0621 (11)	0.0621 (10)	−0.0118 (8)	0.0105 (8)	−0.0079 (9)
C1	0.0488 (11)	0.0440 (10)	0.0508 (10)	0.0030 (8)	−0.0019 (8)	−0.0002 (9)
C2	0.0680 (15)	0.0529 (12)	0.0571 (12)	−0.0071 (10)	−0.0020 (10)	−0.0078 (11)
C3	0.0838 (17)	0.0641 (15)	0.0537 (12)	0.0034 (13)	0.0067 (11)	−0.0091 (11)
C4	0.0675 (15)	0.0632 (14)	0.0598 (13)	0.0122 (11)	0.0133 (11)	0.0077 (12)
C5	0.0653 (15)	0.0559 (13)	0.0884 (17)	−0.0078 (11)	0.0197 (12)	−0.0053 (13)
C6	0.0618 (14)	0.0502 (12)	0.0738 (14)	−0.0067 (10)	0.0130 (11)	−0.0143 (11)
C7	0.0469 (11)	0.0439 (10)	0.0526 (11)	0.0002 (8)	−0.0057 (8)	0.0021 (9)
C8	0.0562 (13)	0.0563 (13)	0.0797 (15)	−0.0096 (10)	0.0048 (11)	−0.0079 (12)
C9	0.0477 (11)	0.0618 (13)	0.0525 (11)	−0.0013 (9)	0.0055 (9)	0.0025 (10)
C10	0.0718 (16)	0.0774 (17)	0.0662 (14)	−0.0118 (13)	0.0018 (12)	−0.0185 (13)

### Geometric parameters (Å, °)

**Table:**

S1—C9	1.743 (2)	C2—H2	0.9300
S1—C10	1.800 (2)	C3—C4	1.387 (4)
S2—C9	1.666 (2)	C3—H3	0.9300
N1—C4	1.378 (3)	C4—C5	1.388 (3)
N1—H1A	0.9008	C5—C6	1.364 (3)
N1—H1B	0.8793	C5—H5	0.9300
N2—C7	1.281 (3)	C6—H6	0.9300
N2—N3	1.381 (2)	C7—C8	1.501 (3)
N3—C9	1.345 (3)	C8—H8A	0.9600
N3—H3N	0.9399	C8—H8B	0.9600
C1—C2	1.390 (3)	C8—H8C	0.9600
C1—C6	1.394 (3)	C10—H10A	0.9600
C1—C7	1.478 (3)	C10—H10B	0.9600
C2—C3	1.383 (3)	C10—H10C	0.9600
			
C9—S1—C10	102.20 (11)	C4—C5—H5	119.4
C4—N1—H1A	112.9	C5—C6—C1	122.2 (2)
C4—N1—H1B	125.7	C5—C6—H6	118.9
H1A—N1—H1B	111.1	C1—C6—H6	118.9
C7—N2—N3	120.32 (17)	N2—C7—C1	114.74 (18)
C9—N3—N2	117.59 (17)	N2—C7—C8	125.9 (2)
C9—N3—H3N	119.2	C1—C7—C8	119.40 (18)
N2—N3—H3N	122.0	C7—C8—H8A	109.5
C2—C1—C6	116.4 (2)	C7—C8—H8B	109.5
C2—C1—C7	123.35 (19)	H8A—C8—H8B	109.5
C6—C1—C7	120.22 (19)	C7—C8—H8C	109.5
C3—C2—C1	121.6 (2)	H8A—C8—H8C	109.5
C3—C2—H2	119.2	H8B—C8—H8C	109.5
C1—C2—H2	119.2	N3—C9—S2	121.66 (16)
C2—C3—C4	121.0 (2)	N3—C9—S1	113.26 (15)
C2—C3—H3	119.5	S2—C9—S1	125.07 (14)
C4—C3—H3	119.5	S1—C10—H10A	109.5
N1—C4—C3	121.6 (2)	S1—C10—H10B	109.5
N1—C4—C5	120.8 (2)	H10A—C10—H10B	109.5
C3—C4—C5	117.5 (2)	S1—C10—H10C	109.5
C6—C5—C4	121.3 (2)	H10A—C10—H10C	109.5
C6—C5—H5	119.4	H10B—C10—H10C	109.5
			
C7—N2—N3—C9	−173.50 (19)	N3—N2—C7—C1	−178.93 (17)
C6—C1—C2—C3	−1.8 (3)	N3—N2—C7—C8	1.7 (3)
C7—C1—C2—C3	178.2 (2)	C2—C1—C7—N2	−177.2 (2)
C1—C2—C3—C4	1.0 (4)	C6—C1—C7—N2	2.9 (3)
C2—C3—C4—N1	−177.5 (2)	C2—C1—C7—C8	2.3 (3)
C2—C3—C4—C5	0.1 (4)	C6—C1—C7—C8	−177.7 (2)
N1—C4—C5—C6	177.3 (2)	N2—N3—C9—S2	−176.19 (15)
C3—C4—C5—C6	−0.3 (4)	N2—N3—C9—S1	5.0 (2)
C4—C5—C6—C1	−0.6 (4)	C10—S1—C9—N3	−176.49 (17)
C2—C1—C6—C5	1.6 (3)	C10—S1—C9—S2	4.73 (19)
C7—C1—C6—C5	−178.4 (2)		

### Hydrogen-bond geometry (Å, °)

**Table:**

*D*—H···*A*	*D*—H	H···*A*	*D*···*A*	*D*—H···*A*
N1—H1A···S2^i^	0.90	2.83	3.722 (3)	170
N3—H3N···S2^ii^	0.94	2.59	3.483 (2)	159
C10—H10A···Cg^iii^	0.96	2.80	3.538 (3)	134

Symmetry codes: (i) *x*−1/2, −*y*+3/2, *z*+1/2; (ii) −*x*+2, −*y*+1, −*z*+1; (iii) −*x*+1, −*y*+1, −*z*+1.
